# Furoshiki traction: broad-area lifting in colorectal endoscopic submucosal dissection with clip-with-line pulley securing

**DOI:** 10.1055/a-2883-1051

**Published:** 2026-09-23

**Authors:** Susumu Banjoya, Ken Ohata, Masaki Koga, Toshihiro Funabiki, Shigenobu Yoshimura, Chiba Hideyuki, Ryoju Negishi

**Affiliations:** 1 Division of Gastroenterology 13635 Itabashi Chuo Medical CenterTokyoJapan; 2 Department of Gastrointestinal Endoscopy 13635 NTT Medical Center TokyoTokyoJapan; 3 Department of Gastroenterology 13635 Omori Red Cross HospitalTokyoJapan

Colorectal endoscopic submucosal dissection (ESD) remains technically challenging. Various traction methods have been developed to improve submucosal visualization; however, most provide traction along a single vector, limiting both exposure and stable maintenance of the dissection plane. 1​ In addition, many methods require intracolonic grasping of a ring and may be complicated by ring slippage when fixation is attempted on the opposite colonic wall. 2​ Although two-dimensional traction systems such as the ATRACT device can provide broader surface traction, they require a dedicated proprietary device and still share the same intracolonic ring-grasping limitation. 3​

We therefore developed “Furoshiki traction,” a two-dimensional traction method based on the CLiPS technique 4​ and ROLM 5​ using only standard through-the-scope clips, without the need for intracolonic ring grasping ( Fig. 1 , Video 1 ).

**Fig. 1:**
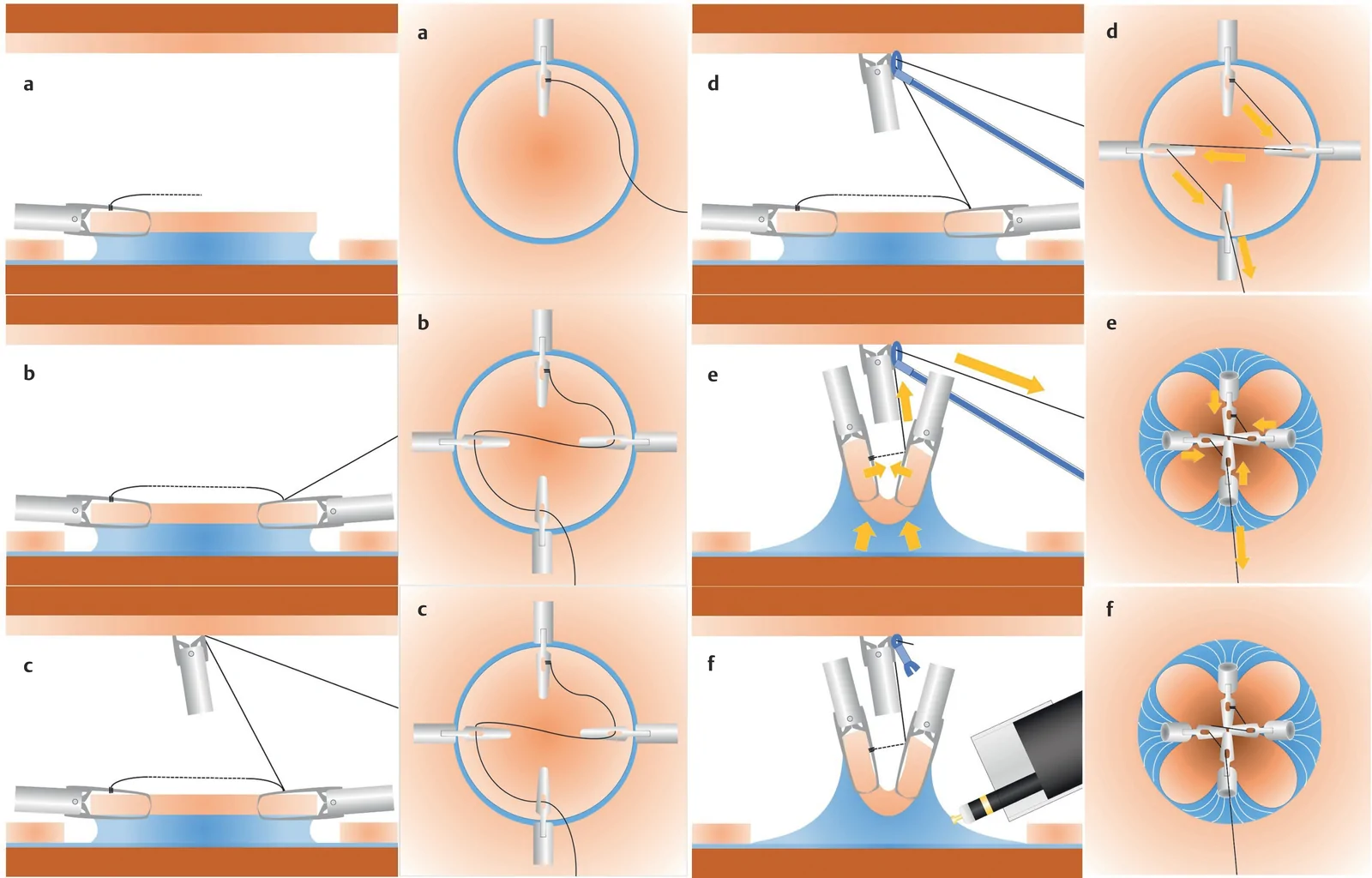
Furoshiki Traction. ( **a** ) Distal clip-with-line placement. ( **b** ) Sequential marginal clip placement with threaded line. ( **c** ) Contralateral colonic wall fixation with clip. ( **d** and **e** ) Traction using the CLiPS technique. ( **f** ) Safe and rapid submucosal dissection.

**Video 1** ESD on a 50-mm cecum using Furoshiki Traction.


A 66-year-old man presented with a 50 mm laterally spreading tumor in the cecum. After circumferential mucosal incision, a SureClip (Micro-Tech, Nanjing, China) preloaded with a suture thread was placed at the lesion margin. The thread was then passed through the hole in the arm of the next clip, which was deployed at an adjacent site. This procedure was repeated sequentially around the lesion periphery. A fixation clip was subsequently placed on the contralateral mucosa, and the thread was tightened using the CLiPS technique, thereby cinching the lesion edges and lifting the entire specimen in a manner resembling a traditional Japanese Furoshiki wrapping cloth. Traction strength was adjusted as needed, and the thread was then secured and cut.

This maneuver elevated the lesion in a tent-like fashion, producing both a flap-lifting effect through bilateral edge elevation and effective countertraction through fixation to the opposite wall. In addition, circumferential convergence reduced the submucosal dissection area geometrically, helping maintain excellent visualization throughout the procedure.

Furoshiki traction enables two-dimensional submucosal traction using only standard equipment, making it a simple, low-cost, and practical method for colorectal ESD.
